# Partial HELLP Syndrome: maternal and perinatal outcome

**DOI:** 10.1590/S1516-31802002000600005

**Published:** 2002-11-01

**Authors:** Joelcio Francisco Abbade, José Carlos Peraçoli, Roberto Antonio Araújo Costa, Iracema de Mattos Paranhos Calderon, Vera Therezinha Medeiros Borges, Marilza Vieira Cunha Rudge

**Keywords:** HELLP syndrome, Partial HELLP Syndrome, Preeclampsia, Maternal outcome, Perinatal outcome, Síndrome HELLP, Síndrome HELLP parcial, Pré-eclâmpsia, Resultados maternos, Resultados perinatais

## Abstract

**CONTEXT:**

HELLP syndrome is a severe complication of pregnancy characterized by hemolysis, elevated liver enzymes and low platelet count. Some pregnant women develop just one or two of the characteristics of this syndrome, which is termed Partial HELLP Syndrome (PHS).

**OBJECTIVE:**

The objective of this study was to evaluate the repercussions on maternal and perinatal outcomes among women that developed PHS and to compare these women with those whose gestational hypertension or preeclampsia did not show alterations for HELLP syndrome in laboratory tests.

**DESIGN:**

Observational, retrospective and analytical study.

**SETTING:**

Maternity Department of Hospital das Clínicas, Faculdade de Medicina de Botucatu, Universidade Estadual Paulista, Botucatu, São Paulo, Brazil.

**SAMPLE:**

Pregnant or post-delivery women who had a blood pressure elevation that was first detected after mid-pregnancy, with or without proteinuria, between January 1990 and December 1995.

**MAIN MEASUREMENTS:**

Analysis was made of maternal age, race, parity, hypertension classification, gestational age at the PHS diagnosis, alterations in laboratory tests for HELLP syndrome, time elapsed to discharge from hospital, maternal complications, mode of delivery, incidence of preterm birth, intrauterine growth restriction, stillborn and neonatal death.

**RESULTS:**

Three hundred and eighteen women were selected; forty-one women (12.9%) had PHS and 277 of them (87.1%) did not develop any of the alterations of the HELLP syndrome diagnosis. Preeclampsia was a more frequent type of hypertension in the PHS group than in the hypertension group. None of the women with isolated chronic hypertension developed PHS. The rate of cesarean delivery, eclampsia, and preterm delivery was significantly greater in the PHS group than in the hypertension group.

**CONCLUSION:**

We observed that aggressive procedures had been adopted for patients with PHS. These resulted in immediate interruption of pregnancy, with elevated cesarean rates and preterm delivery. Such decisions need to be reviewed, in order to reduce the cesarean rate and the incidence of preterm delivery.

## RESUMO

**CONTEXTO:**

A síndrome HELLP é uma grave complicação da gestação caracterizada por hemólise, elevação das enzimas hepáticas e plaquetopenia. Algumas gestantes desenvolvem somente uma ou duas dessas características da síndrome HELLP. Esse quadro é denominado de síndrome HELLP parcial (SHP).

**OBJETIVO:**

O objetivo deste estudo foi avaliar as repercussões maternas e perinatais das mulheres que desenvolveram SHP e comparar os resultados com mulheres que tiveram hipertensão gestacional ou pré-eclâmpsia sem alterações dos exames laboratoriais para síndrome HELLP.

**TIPO DE ESTUDO:**

Observacional, retrospectivo e analítico.

**LOCAL:**

Maternidade do Hospital das Clínicas da Faculdade de Medicina de Botucatu, Uni-versidade Estadual Paulista, Botucatu, São Paulo, Brasil.

**AMOSTRA:**

Foram selecionadas gestantes ou puérperas que tiveram elevação dos níveis pressóricos detectada pela primeira vez após a primeira metade da gestação com ou sem proteinúria entre janeiro/1990 a dezembro/ 1995. As mulheres foram divididas em dois grupos: Grupo SHP – quando as mulheres com hipertensão arterial tinham pelo menos uma, mas não todas as alterações de exames que demonstravam hemólise, elevação das enzimas hepáticas ou plaquetopenia – e Grupo Hipertensas – pacientes com hipertensão sem alterações nos exames laboratoriais para síndrome HELLP.

**PRINCIPAIS VARIÁVEIS:**

Analisamos idade materna, raça, paridade, classificação da hi-pertensão, idade gestacional no diagnóstico da SHP, alterações nos exames laboratoriais para síndrome HELLP, tempo de perma-nência no hospital, complicações maternas, via de parto, incidência de prematuridade, restrição de crescimento intra-uterino, natimortos e neomortos.

**RESULTADOS:**

318 mulheres foram selecionadas, das quais 41 (12,9%) tiveram SHP e 277 (87,1%) não desenvolveram alterações dos exames laboratoriais que compõem o diagnóstico da síndrome HELLP. A pré-eclâmpsia foi um tipo de hipertensão mais freqüente no grupo SHP que no grupo hipertensas. Não houve pacientes com hipertensão crônica isolada que desenvolveram SHP. A taxa de cesárea, eclâmpsia e de partos prematuros foi significativamente mais freqüente no grupo SHP que no grupo hipertensas.

**CONCLUSÃO:**

Observamos uma conduta agres- siva nas pacientes com SHP, que resultou na interrupção imediata da gestação, com elevada taxa de cesárea e de recém-nascido pré-termo. Esta conduta deve ser revista para a redução desses índices.

## INTRODUCTION

Hemolysis, elevated liver enzymes and low platelet count are alterations in laboratory tests that are found in pregnant or post-delivery women who have preeclampsia. The term *HELLP syndrome* was coined for this set of alterations by Weinstein in 1982.^1^ Since then many reports have been presented, but the quantification of laboratory tests has differed among them.^2-15^

Since Sibai^2^ proposed strict criteria for the diagnosis of the “true HELLP syndrome” it has been observed that many women with severe preeclampsia may have laboratory abnormalities such as isolated hemolysis or low platelet count or elevated liver enzymes, without the complete HELLP syndrome. Women with partial HELLP syndrome (PHS) should be studied and managed separately from women with HELLP syndrome or severe preeclampsia.^15^

The incidence of HELLP syndrome is 2 to 12%,^8,11,16-21^ while the incidence of PHS is unclear, but probably around 21 to 24%.^15,22^ There is no information about PHS incidence in Brazil.

HELLP syndrome may begin as PHS, because it is an insidious and progressive disease. This characteristic is corroborated by the different elapsed times seen in laboratory tests for its alterations and the progress of the disease. Another factor that supports this idea is that in spite of delivery being the definitive treatment for women with HELLP syndrome, the condition of some women worsens over the first 48 hours after delivery.^23^

The purpose of this report was to compare maternal and perinatal outcomes between women with PHS and women who had severe blood pressure elevation but normal laboratory tests for HELLP syndrome.

## METHODS

This was a retrospective, observational and analytic study. It was made in the Maternity Department of a university hospital, Hospital das Clínicas of Universidade Estadual Paulista, which is a third-level public hospital located in the central region of São Paulo State, Brazil. We searched through the perinatal database of our Maternity Department for pregnant or post-delivery women who had had a blood pressure elevation that was first detected after mid-pregnancy, either with proteinuria (preeclampsia) or without it (gestational hypertension) between January 1991 and December 1995. We reviewed maternal and neonatal medical charts.

HELLP syndrome was defined by the presence of all of the three following criteria: hemolysis (characteristic peripheral blood smear, serum lactate dehydrogenase ≥ 600 U/ l, total serum bilirubin ≥ 1.2 mg/ml), elevated liver enzymes (serum aspartate aminotransferase ≥ 70 U/l), and low platelet count (< 100,000/µl). Partial HELLP syndrome (PHS) was defined by the presence of one or two features of HELLP but not the complete syndrome.^15^ Patients were defined as having severe hypertension according to the criteria of the National High Blood Pressure Education Program (2000).^24^ The patients were divided into two groups: *Partial HELLP Syndrome Group* (patients with PHS) and *Hypertension Group* (patients with severe gestational hyper- tension/preeclampsia but without alterations in laboratory tests for HELLP syndrome). Women with renal, liver or hematological disease and multiple pregnancy were excluded from the study.

Gestational age was determined by using the best-accepted obstetric criteria, including menstrual history, early clinical evaluation and ultrasonography at < 20 weeks of gestation. The classification of the hypertensive disorders of pregnancy was done according to the National High Blood Pressure Education Program (2000),^24^ i.e. chronic hypertension, gestational hypertension, preeclampsia/eclamp-sia, gestational hypertension or preeclampsia superimposed upon chronic hypertension. We evaluated the abnormal laboratory findings in the PHS group and the gestational age at which PHS was diagnosed. We compared the time elapsed until discharge from hospital, maternal complications (imminent eclampsia, eclampsia, abruptio placentae and maternal mortality), mode of delivery (cesarean section), preterm delivery (gestational age < 37 weeks), perinatal outcome (intrauterine growth restriction, stillborn and neonatal death).

Data are presented as incidences. Statistical comparisons were performed by χ^2^ analysis, Pearson χ^2^ analysis and exact Fisher test, as appropriate. A *p* value < 0.05 was considered significant. Statistical analysis was performed using the Statistical Package in Social Science for Windows^®^ (SPSS Inc, Chicago, version 10.0).

The procedures above were in accordance with the ethical standards of the Medical Ethics Committee of our university and with the Declaration of Helsinki.^25^

## RESULTS

During the study period, 329 patients had clinical and laboratory findings of severe hypertension. Six women were excluded from the analysis because they had complete HELLP syndrome. Three women had multiple pregnancy and two had chronic renal insufficiency, and they were also excluded.

Among the remaining 318 women, 41 (12.9%) had PHS and 277 (87.1%) had elevated blood pressure levels, clinical and laboratory findings of severe gestational hypertension (GH) or preeclampsia (PE), with normal laboratory test results for HELLP syndrome. Demographic characteristics are presented in Table 1. Most of the women were in the 19 to 34-year-old age range. White women were more frequent in both groups. Twenty-one women (51.2 %) were nullipara in the PHS group and 128 (46.2%) in the hypertension group. There was no significant difference in these variables between the groups.

**Table 1:** Baseline characteristics of women with partial HELLP syndrome (hemolysis, elevated liver enzymes and low platelets) and with only hypertension, according to maternal age, race, parity and hypertension classification

	Partial HELLP Syndrome Group		Hypertension Group		*p - value*
	N	%	N	%	
**Maternal age (years)**					*0.305*
<19	6	14.6	50	18.1	*-*
19 to 34	24	58.5	180	64.9	*-*
≥ 35	11	26.8	47	17.0	*-*
**Race**					*0.915*
White	33	80.5	220	79.4	*-*
Black	8	19.5	57	20.6	*-*
**Parity**					*0.858*
Nullipara	21	51.2	131	47.3	*-*
1 to 4	18	43.9	128	46.2	*-*
≥ 5	2	4.9	18	6.5	*-*
**Classification of hypertension**					*0.306*
Gestational hypertension	12	29.2	103	37.2	-
Preeclampsia	17	41.5	82	29.6	-
Gestational hypertension superimposed upon chronic hypertension	8	19.5	45	16.2	-
Preeclampsia superimposed upon chronic hypertension	4	9.8	47	17.0	-

Among these women, 9.8% had preeclampsia and 19.5% had gestational hypertension superimposed upon chronic hypertension. Isolated preeclampsia was more frequent in the PHS group (41.5%) than in the hypertension group (29.6%). Gestational hypertension was observed in 13 women (29.3%) in the PHS Group and 103 (37.2%) in Hypertension Group. There were no significant differences among these factors (Table 1).

PHS was diagnosed mainly in preterm pregnancies (66.7%). Twenty-two women (56.4%) had gestations of less than 34 weeks and 5 of them (12.8%) had gestations of less than 29 weeks. Of the 41 patients with PHS, 14 (34.1%) only had hemolysis, 7 (17.1%) had hemolysis and low platelet count and 5 (12.2%) had hemolysis and elevated liver enzymes. Eight women (19.5%) only had low platelet count, 4 (9.8%) had low platelet count and elevated liver enzymes. Elevated liver enzymes alone was observed in 3 patients (7.3%) (Table 2).

**Table 2:** Distribution of partial HELLP syndrome (PHS) group (hemolysis, elevated liver enzymes and low platelets), according to gestational age at which PHS was diagnosed and type of alterations seen in laboratory tests for HELLP syndrome

	Partial HELLP Syndrome Group	
	N	%
**Gestational age at which PHS was diagnosed**		
< 37 weeks	26	66.7
23 – 28 weeks	5	12.8
29 – 34 weeks	17	43.6
35 – 36 weeks	4	10.3
**Alterations seen in laboratory tests for HELLP syndrome**		
Hemolysis	14	34.1
Low platelet count	8	19.5
Elevated liver enzymes	3	7.3
Hemolysis + Low platelet count	7	17.1
Hemolysis + Elevated liver enzymes	5	12.2
Low platelet count + Elevated liver enzymes	4	9.8

There was a significant difference in the length of time spent in hospital between the groups (greater than or less than four days). Thirty-six women (87.8%) with PHS and 202 of the hypertension group (74.0%) stayed in hospital for at least four days (Table 3).

**Table 3:** Distribution of women with partial HELLP syndrome (hemolysis, elevated liver enzymes and low platelets) and with only hypertension, according to time elapsed to discharge from hospital, maternal complications (abruptio placentae, imminent eclampsia, eclampsia, maternal death) and mode of delivery

	Partial HELLP Syndrome Group		Hypertension Group		*p - value*
	N	%	N	%	
Time elapsed to discharge from hospital					*0.036*
< 4 days	5	12.2	72	26.0	*-*
≥ 4 days	36	87.8	205	74.0	*-*
Maternal complications					*-*
Imminent eclampsia	18	43.9	95	34.9	*0.153*
Eclampsia	6	14.6	16	5.8	*0.048*
Abruptio placentae	0	0.0	13	4.7	*-*
Maternal death	0	0.0	1	0.4	*-*
Mode of delivery					*0.042*
Vaginal	4	9.8	62	22.4	*-*
Cesarean	37	90.2	215	77.6	*-*

There was no difference in the incidence of abruptio placentae, imminent eclampsia and maternal death. Eclampsia was more frequent in the PHS group (14.6%) than in the hypertension group (5.8%) (Table 3), with a significant difference between the two groups.

The mode of delivery is shown in Table 3. The overall cesarean delivery rate was significantly higher in the PHS group (90.2%) than in the group with normal laboratory values for HELLP syndrome (77.6%).

Despite the incidence of preterm delivery being higher in the PHS group (70.7%) than in the hypertension group (57.7%), and the incidence of stillbirths being higher in the hypertension group (5.1%) than in the PHS group (0.0%), no statistically significant difference was observed. There were no differences in the incidence of intrauterine growth restriction and neonatal death (Table 4).

**Table 4:** Distribution of women with partial HELLP syndrome (hemolysis, elevated liver enzymes and low platelets) and with only hypertension, according to incidence of preterm delivery, stillborn, neonatal death and intrauterine growth restriction

	Partial HELLP Syndrome Group		Hypertension* Group*		*p - value*
	N	%	N	%	
Preterm delivery	29	70.7	158	57.7	*0.760*
Term delivery	12	29.3	116	42.3	
Neonatal death	3	7.3	24	8.8	*0.309*
Stillbirth	0	0.0	14	5.1	*-*
Birth live	38	92.7	236	86.1	
Intrauterine growth restriction	11	26.8	75	27.4	*0.897*

## DISCUSSION

Although the term HELLP syndrome was not coined until 1982,^1^ its pathological features have been recognized for at least 100 years.^26^ However, controversies persist regarding the diagnosis, management, and prognosis of this enigmatic disease. This uncertainty exists partly because its pathophysiological mechanism remains obscure and partly because of disagreement about the criteria used to define this syndrome.

Sibai^2^ defined standardized strict laboratory criteria for disease diagnosis, which have been used in this study to define the group of women with HELLP syndrome. Other, previous authors used less strict criteria, consequently including in their studies women who we would have considered to have only partial HELLP syndrome.

HELLP syndrome or PHS can be diagnosed during pregnancy or after delivery in women whose blood pressure elevation was first detected after mid-pregnancy, either with or without proteinuria. Despite many authors having shown that HELLP syndrome is a complication ofpreeclampsia or eclampsia, Sibai^2^ and Martin et al.^23^ observed that hypertension and proteinuria may be absent or only slight. Even though HELLP syndrome is considered to be a variant or an atypical variant form of severe preeclampsia, its severity is reflected in its laboratory parameters, and not in the usual clinical parameters of blood pressure and proteinuria that typically reflect preeclampsia disease se-verity.^27^ We observed that 48.7% of women did not have proteinuria. It confirms the idea that PHS can occur among women with gestational hypertension or gestational hypertension superimposed upon chronic hypertension. Thus, some of these patients may have a variety of signs and symptoms, none of which are diagnostic of classic severe preeclampsia.

PHS can progress to HELLP syndrome because the alterations seen in laboratory tests may take place after different elapsed times.^20^ Audibert et al.^15^ did not observe disseminated intravascular coagulation or other maternal and perinatal complications among women with PHS or severe preeclampsia. This information suggests that women with PHS have some complications but they are not as severe as in HELLP syndrome. It emphasizes the importance of recognizing HELLP syndrome as a distinct entity that is associated with serious maternal morbidity.

We believe that the management of women with PHS must be different from the management of women with severe preeclampsia or HELLP syndrome. This may be achieved by clinical management and it may not be necessary to interrupt the pregnancy, since the maternal and perinatal outcomes among women with PHS did not exhibit any differences in comparison with women with severe gestational hypertension or preeclampsia, except for the incidence of eclampsia.

Preeclampsia increases the cesarean rate, which ranges from 29.6 to 55.0%,^28-32^ and this incidence is always significantly higher than the incidence of cesareans among healthy pregnant women or pregnant women with isolated chronic hypertension. The cesarean rate among pregnant women with hypertension is very high in Brazil. This rate can reach 76.7%,^33-35^ and it is similar to the incidence in the hypertension group of our study (77.6%).

The cesarean rate in the PHS group was very high, because when the disease was diagnosed we opted for the interruption of the pregnancy, so as to avoid evolution from PHS to HELLP syndrome and worsening of the maternal and perinatal outcomes. Audibert et al.^15^ and Abramovici et al.^22^ showed elevated cesarean rates among women with PHS, 36% and 54% respectively, but these rates were lower than the rate in the PHS group in our study (90.3%). This means that we should not indicate immediate delivery by cesarean section for almost all women with PHS, but try to encourage the conservative management of these patients.

Gestational hypertension and preeclampsia must be diagnosed as soon as possible, so as to get the best maternal and perinatal outcomes. Consequently, it is recommended that all pregnant or post-delivery women with slight or severe blood pressure elevation should have a complete blood cell, platelet count and liver enzyme determination, in order to make an early diagnosis of PHS or HELLP syndrome.

## CONCLUSION

HELLP syndrome in pregnant or postdelivery women with gestational hypertension or preeclampsia needs to be diagnosed as early as possible. But in cases with a diagnosis of partial HELLP syndrome, we observed that aggressive procedures had been adopted. These resulted in immediate interruption of pregnancy, with elevated cesarean rates and preterm delivery. Such decisions need to be reviewed and a management strategy of monitoring could be attempted, in order to improve perinatal and maternal outcomes.
